# Cognitive load recognition in simulated flight missions: an EEG study

**DOI:** 10.3389/fnhum.2025.1542774

**Published:** 2025-03-05

**Authors:** Yueying Zhou, Xijia Xu, Daoqiang Zhang

**Affiliations:** ^1^School of Mathematics Science, Liaocheng University, Liaocheng, China; ^2^Key Laboratory of Brain-Machine Intelligence Technology, Ministry of Education, Nanjing University of Aeronautics and Astronautics, Nanjing, China; ^3^Department of Psychiatry, Affiliated Nanjing Brain Hospital, Nanjing Medical University, Nanjing, China; ^4^College of Artificial Intelligence, Nanjing University of Aeronautics and Astronautics, Nanjing, China

**Keywords:** electroencephalogram (EEG), cognitive load recognition, simulated flight, convolutional neural network, brain-computer interfaces (BCI)

## Abstract

Cognitive load recognition (CLR) utilizing EEG signals has experienced significant advancement in recent years. However, current load-eliciting paradigms often rely on simplistic cognitive tasks such as arithmetic calculations, failing to adequately replicate real-world scenarios and lacking applicability. This study explores simulated flight missions over time to better reflect operational environments and investigate temporal dynamics of multiple load states. Thirty-six participants were recruited to perform simulated flight tasks with varying cognitive load levels of low, medium, and high. Throughout the experiments, we collected EEG load data from three sessions, pre- and post-task resting-state EEG data, subjective ratings, and objective performance metrics. Then, we employed several deep convolutional neural network (CNN) models, utilizing raw EEG data as model input, to assess cognitive load levels with six classification designs. Key findings from the study include (1) a notable distinction between resting-state and post-fatigue EEG data; (2) superior performance of shallow CNN models compared to more complex ones; and (3) temporal dynamics decline in CLR as the missions progressed. This paper establishes a potential foundation for assessing cognitive states during intricate simulated tasks across different individuals.

## 1 Introduction

Cognitive load recognition is a crucial paradigm and common application of passive brain-computer interfaces (BCI), as well as a significant aspect of intelligent human-computer interaction (Arico et al., 2017; Debie et al., 2021; Heard et al., 2018; Dimitrakopoulos et al., 2017). It serves as the crucial link in cognitive state perception and forms the foundation for cognitive emotion monitoring, exploration, and passive cognitive adjustment. Cognitive load can be defined as the consumption of mental resources or the occupation of brain information processing during human-computer interaction tasks (Wickens, 2002; Zhou et al., 2022). The level of cognitive load directly affects the efficiency, accuracy, and safety of task execution. Prolonged exposure to excessive cognitive load can lead to a range of physical and mental health issues, posing significant hidden risks to the safety of human-computer interaction tasks, and potentially resulting in human factor accidents (Shao et al., 2024; Zhou et al., 2022; Yan et al., 2023; Wang et al., 2024). Hence, in highly secure and complex human-computer interaction systems that require high cognitive abilities from operators (such as simulated flight tasks and aviation control), it is essential to decipher and provide feedback on human cognitive load status (Zhou et al., 2023). This enables dynamic task allocation, issuance of overload warnings, enhancement of emotional experiences, improvement of system usability, and operational efficiency and safety (Dell’Agnola et al., 2022; Appel et al., 2023).

Electroencephalogram (EEG) signals have gained widespread adoption in passive BCI research for decoding purposes due to their sensitive capacity to capture the brain’s original electrophysiological response, high temporal resolution, and relatively low acquisition and processing costs (Gong et al., 2024; Biasiucci et al., 2019). They are currently considered one of the most frequently utilized brain signals (Zhang et al., 2019).

The majority of current research for cognitive load recognition (CLR) revolves around simple cognitive tasks, such as *n*-back (Zanetti et al., 2021), English letter memory (Zhou et al., 2022), and mathematical computation (Kakkos et al., 2021). While these tasks demonstrate high repeatability and prompt cognitive state responses, they have less emphasis on recognizing cognitive load in complex and dynamic real-world task scenarios (Chen et al., 2023), thus limiting their practical applicability. Therefore, this study aims to explore the recognition of cognitive load under simulated flight missions to provide more effective strategies and tools for managing cognitive load in specific task environments.

**Figure 1:**
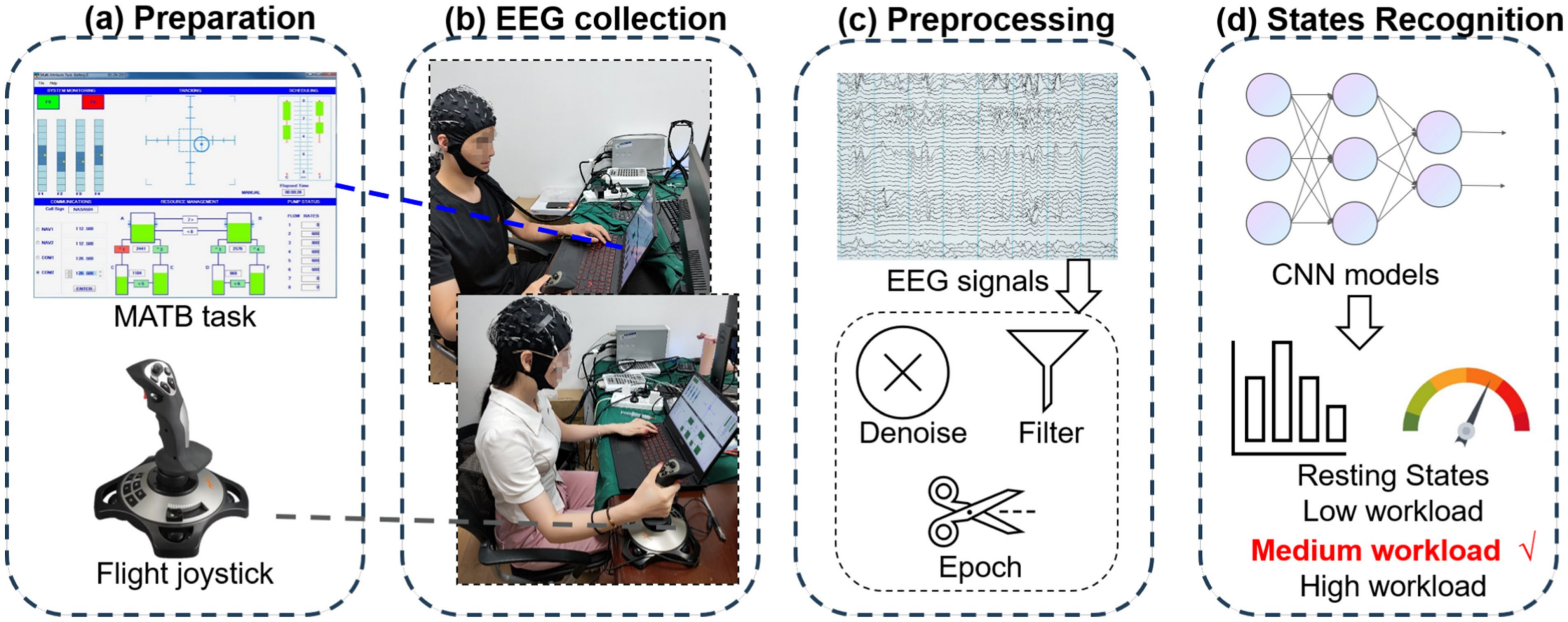
Overview of the MATB load recognition task, including **(a)** preparation, **(b)** EEG collection, **(c)** EEG preprocessing, and **(d)** load states recognition.

To achieve this goal, we recruited a group of participants who underwent three sets of simulated flight tasks Multi-Attribute Task Battery (MATB) (Santiago-Espada et al., 2011; Cegarra et al., 2020), involving eliciting low, medium, and high levels of cognitive load. The overview of the MATB load recognition task is shown in Figure 1. The decoding process of cognitive load based on EEG involves several steps: (a) designing an experiment to induce cognitive load using EEG, (b) acquiring EEG data, (c) preprocessing the EEG signals, and (d) constructing decoding models utilizing machine learning techniques, and subsequently recognizing the cognitive load state of the brain.

By collecting resting-state EEG data from participants before and after the tasks, along with subjective evaluation ratings and objective task performance data, we conducted a comprehensive evaluation of operators’ cognitive load levels. Recently, convolutional neural networks (CNNs) have been specifically designed and extensively validated in the fields of active and passive EEG signal decoding tasks, and have demonstrated interpretable and satisfactory decoding performance (Zhou et al., 2022; Jiao et al., 2018; Qiao and Bi, 2020; Lin et al., 2024). For example, Jiao et al. (2018) introduced a combined model that integrates CNNs with the Restricted Boltzmann Machine to effectively utilize both spectral and temporal knowledge of EEG time series, where deep CNN architecture comprised four convolutional layers and two fully connected layers. Qiao and Bi (2020) proposed a deep hybrid network that integrates a CNN and a Bidirectional Neural Turing Machine for cognitive state evaluation. The CNN focuses on maintaining spatial-spectral characteristics of EEG signals. Meanwhile, the Bidirectional Neural Turing Machine is intended to capture temporal representations from the features provided by the CNN. Lin et al. (2024) developed a transfer CNN with depthwise separable convolution to mine the EEG pattern consisting of rhythmic energies over time.

Besides, some compact and reproducible CNN models have been optimized for considering the spatiotemporal characteristics of EEG signals, such as shallow CNN (Schirrmeister et al., 2017), EEGNet (Lawhern et al., 2018), EEGNex (Chen et al., 2024), and EEGTCN (Ingolfsson et al., 2020). Specifically, EEGNet (Lawhern et al., 2018) utilizes a convolutional structure tailored for processing temporal EEG data, whereas EEGNex (Chen et al., 2024) improves its sensitivity towards EEG events by effectively extracting spatial representations. EEGTCN (Ingolfsson et al., 2020), on the other hand, merges temporal convolutions with conventional CNN structures to efficiently capture temporal features in EEG signals. These specialized CNN models for EEG data offer robust tools for EEG classification and decoding tasks. Inspired by the above research, we employed several end-to-end deep CNN models based on raw EEG data to classify the six cognitive load tasks and explore the EEG signal features under different cognitive load states.

During the analysis of the experimental results, several notable findings emerged. Firstly, there was a clear differentiation between resting-state data and fatigued resting-state data, indicating distinct EEG signal patterns across different cognitive load states. Secondly, shallow CNN model exhibited better performance in task classification, offering new insights into processing complex EEG data. Finally, as the tasks progressed, the accuracy of classifying the three sets of simulated flight tasks showed a decreasing trend, likely reflecting cognitive fatigue among operators during prolonged high cognitive load tasks. These findings have practical implications for recognizing cognitive load in specific operators and designing relevant cognitive tasks. They also provide new research perspectives and methods for EEG-based CLR. The contributions of this study include:

1. Delving deeply into more complex cognitive tasks fills a gap in the design of existing simple numerical tasks.
2. Collecting load task EEG data, resting-state EEG data, subjective self-rating scales, and objective task data through experiments provides a multi-faceted source of information for MATB task research.
3. Employing the end-to-end deep CNN models derived from raw EEG data to categorize cognitive load, which broadens the scope of methods for identifying operators’ cognitive load.
4. Identifying distinct characteristics between resting-state data and fatigue-induced resting-state data during task performance, as well as performance disparities among various deep CNN models.

The remainder of the paper is structured as follows. Section 2 details materials and methods, encompassing load-elicitation paradigm, EEG collection, and the design of recognizing load across time. Section 3 presents the experimental results, including subjective scale results, objective behavior analysis, and CLR outcomes. Section 4 delves into brain activation patterns and data visualization, and gives the limitations and future outlook. Lastly, we conclude the whole paper.

## 2 Materials and methods

This section presents information on recruited subjects, the load-evoked experimental paradigm, EEG data acquisition and preprocessing, and the approaches adopted for identifying cognitive load, using both traditional classifiers and advanced CNN-based models.

### 2.1 Subjects

We have recruited a total of 36 college students with science majors (18 women, average age: 23.2 ± 1.7 years) from Nanjing University of Aeronautics and Astronautics. All participants were right-handed except for two participants. They had no prior experience in flight simulation. They had normal or corrected vision and reported no factors inducing anxiety or fatigue. Before the experiment commenced, participants were asked to abstain from alcohol consumption and caffeine and to ensure 8 h of sleep. Also, we explained the specific experimental process to all subjects and obtained the informed consent of each subject. The study was approved by the Ethics Committee of Affiliated Nanjing Brain Hospital, Nanjing Medical University. To mitigate fatigue, subjects were provided rest periods following each task.

### 2.2 Load-elicited paradigm

#### 2.2.1 MATB task

**Figure 2:**
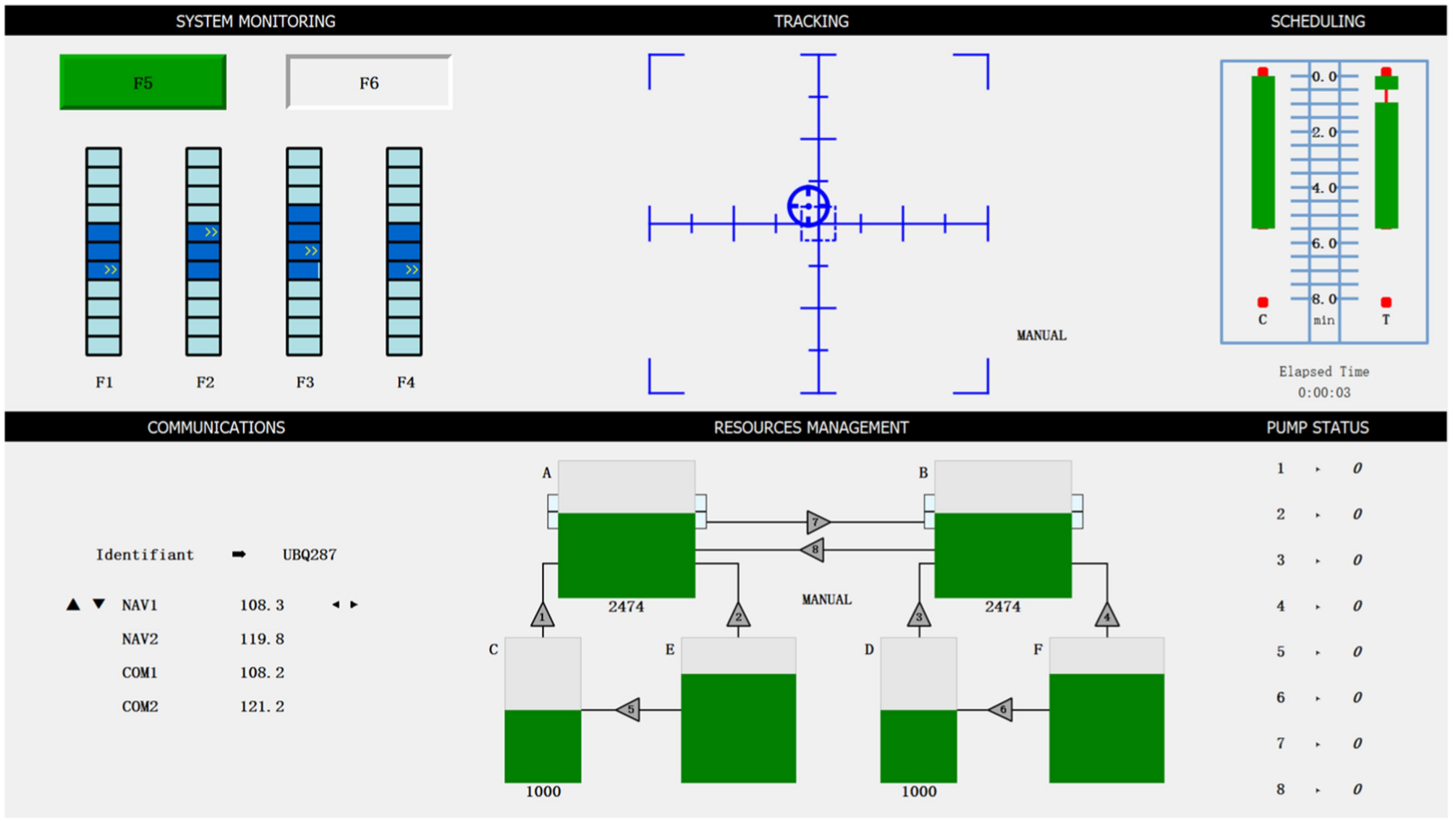
Screenshot of the OpenMATB.

We have used the OpenMATB software (Cegarra et al., 2020), an open-source adaptation of the original MATB task, to perform the MATB II task. The task requires participants to engage in multiple concurrent activities displayed on the screen. The task used in this study consists of three primary components: system monitoring task, target tracking, and fuel resource management, each simulating different operations performed by pilots during aircraft flight. The paradigm design is shown in Figure 2.

In Figure 2, the system monitoring task is situated in the upper left. It simulates the monitoring of instrument indications during a flight mission. It requires participants to oversee six visual indicators (including two warning lights and four cursor pointers) and correct any abnormal behavior. The target tracking task is located at the upper center of Figure 2. In this task, participants engage in a manual simulation where they are required to maintain the target within the aircraft’s tracking range to the best of their ability. The resource management task is positioned in the lower central portion of Figure 2, consisting of six fuel tanks and eight valves. Participants are required to develop a reasonable fuel management strategy to keep the fuel volume of two main fuel tanks A and B at 2,500 gallons.

**Figure 3:**
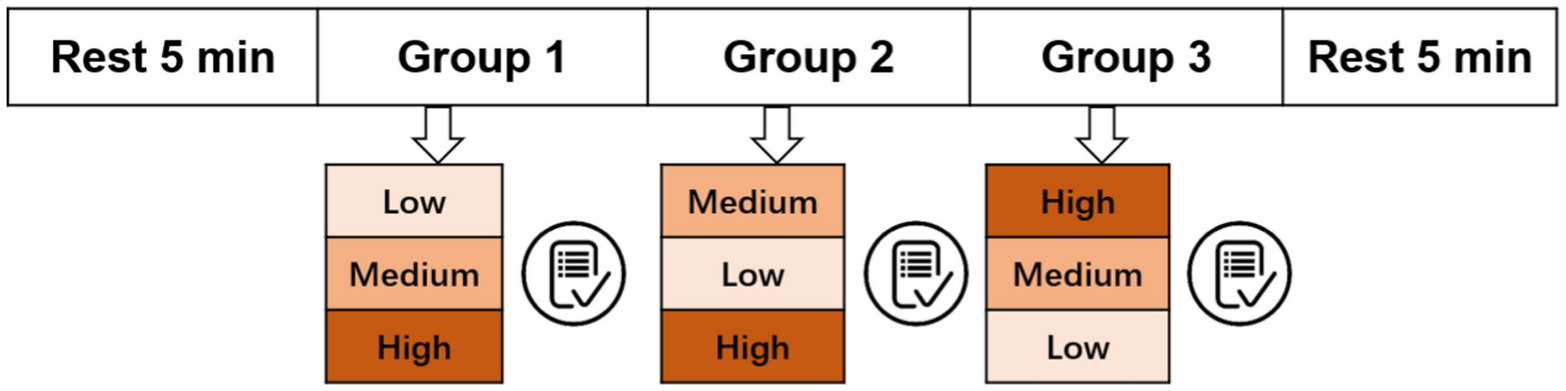
The execution process of the MATB task.

Before the experiment started, participants were informed of the specific procedures and details of the experiment, and participants were asked to conduct a pre-experiment until they were familiar with the experimental procedures. The procedure for executing the entire task is illustrated in Figure 3. In the formal experiment, each subject completes two types of tasks. The first was the resting state task, including a pre-task of 5 min and a post-task of 5 min. The second was the task that induced cognitive load data with three groups.

During the resting states task, participants are required to watch the computer screen with a MATB screen capture (static image) for 5 min. For three groups of MATB tasks, each group of load-inducing tasks includes three difficulty levels of eliciting low, medium, and high loads (depicted in Figure 3 with different shades of orange), with each level lasting for 5.5 min, and the order within each group is different. The difficulty of a task is contingent upon the inherent difficulty of the task itself and the number of concurrent tasks that are being processed.

#### 2.2.2 Task design

The task parameter settings of MATB II under different difficulty conditions are shown in Table 1. The overall difficulty is modulated by adjusting the difficulty levels of individual subtasks. System monitoring and resource management tasks can be interacted with using the keyboard and mouse. They can regulate mental load by adjusting the frequency of abnormal events. The tracking task is controlled with a flight joystick and regulates load by adjusting the tracking trajectory of the center rectangle. We use the PXN-2113PRO flight joystick (Shenzhen PXN Electronics Technology Co., Ltd.) in our experiment, which can be seen in Figure 1a.

**Table 1:** Load-elicited task design.

Subtask	Low	Medium	High
System monitoring	27–40 s/time, 10 times	8–13 s/times, 30 times	4–7 s/times, 50 times
Tracking	Trajectory range 2 cm	Trajectory range 1.5 cm	Trajectory range 1 cm
Resource management	24–40 s/time, 10 times	14–20 s/time, 20 times	7–13 s/time, 30 times

As shown in Table 1, for the low-load-elicitation design, we interact with system monitoring with 10 times abnormal events lasting with 27–40 s, tracking trajectory range with 2 cm, and resource management task with 10 times abnormal events lasting with 24–40 s. For the medium-load-elicitation design, we interact system monitoring with 30 times abnormal events lasting 8–13 s, tracking trajectory range with 1.5 cm, and resource management task with 20 times abnormal events lasting 14–20 s. For the high-load-elicitation design, we interact system monitoring with 50 times abnormal events lasting 4–7 s, tracking trajectory range with 1 cm, and resource management task with 30 times abnormal events lasting 7–13 s.

During the experiment, subjects are requested to complete the subjective NASA-TLX scale (Hart, 1988) promptly. Data pertaining to task performance are simultaneously recorded, including the reaction time for system monitoring task, the standard deviation of fuel level for resource management task, and the tracking error for tracking task.

### 2.3 EEG collection and preprocessing

During the EEG acquisition, Neuroscan (Compumedics, Australia), a high-precision wired EEG acquisition and analysis system, was used to collect 64 channels of EEG data of subjects. It includes the Neuroscan SynAmps2 amplifier and CURRY8 acquisition software. The electrode distribution follows the 10–20 international standard. The grounding electrode is positioned between the FPz and Fz electrodes on the EEG cap, with the reference electrode situated at the M2 point on the right mastoid. The acquisition frequency was 2,000 Hz and the impedance of all electrodes was maintained below 20 kΩ.

We apply a simple preprocessing pipeline to preprocess the raw EEG time series. First, we have used CURRY8 software to perform preliminary preprocess. To be specific, we use VEOG to remove the eye-blink artifacts and then average the data. Afterward, we used Matlab 2019a and EEGLAB plug-in (Delorme and Makeig, 2004) to further preprocess the data, including the following steps. VEO and trigger channels are removed, remaining 62 EEG channels. The remained EEG data are referenced to the average electrodes. Next, a band-pass filter is applied between 0.1 to 70 Hz, and a 50 Hz power line filter is also applied. These steps help maintain useful information and ease the power line noise. Subsequently, the EEG data are decimated to 250 Hz to decrease the model’s calculation cost. Then, the downsampled EEG data are segmented into nine 5.5 min subgroup data for task states (two 5 min for resting states) from −1 to 330 s (−1 to 300 s for resting states) after the stimulus starts. Finally, the 2 s epoch is extracted from the corresponding subgroup data. As such, each subgroup of load task of each subject has 165 EEG trials, and each subgroup of resting states has 150 EEG trials. Thus, the model input has the array shape of 62 channels × 500 time points in 2 s. However, due to the operation error, we have removed the EEG data of Group 3 of subject # 13.

### 2.4 Load classification

**Figure 4:**
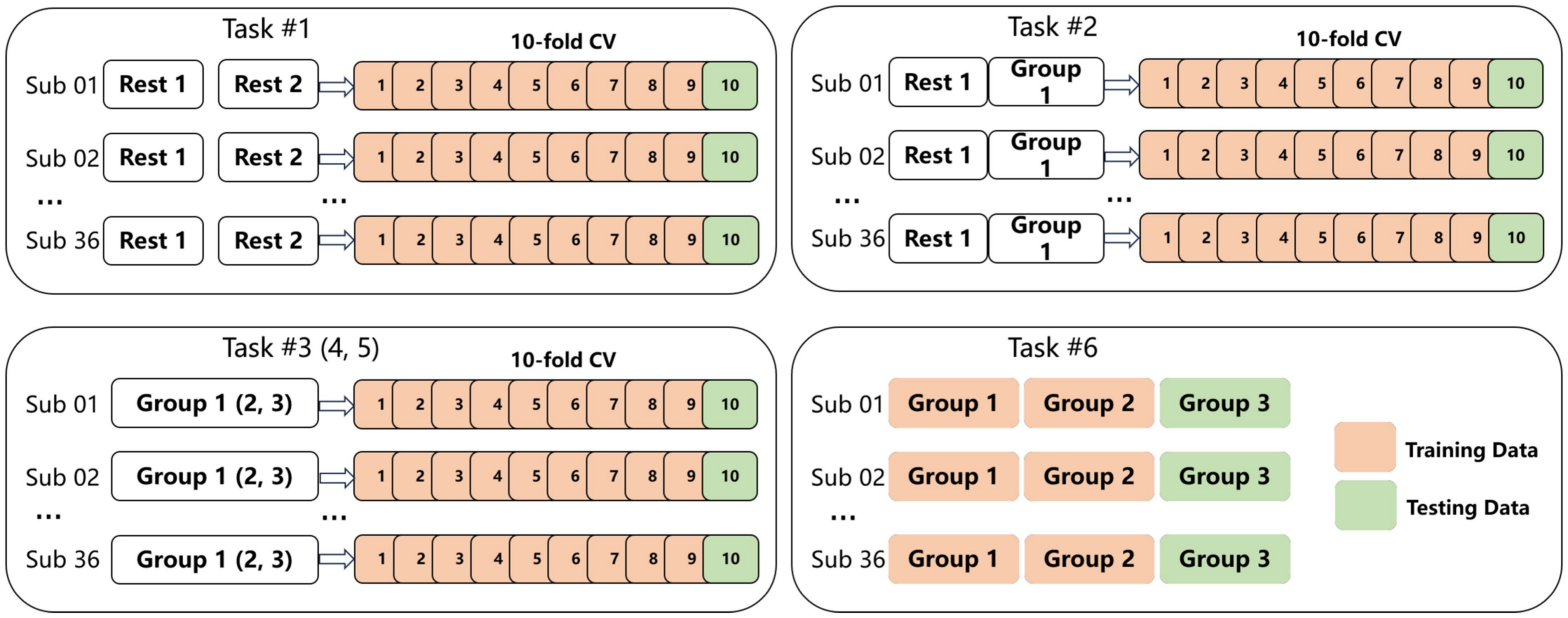
Six CLR decoding tasks in this paper.

We design six load recognition tasks, as shown in Figure 4.

Task #1: Binary classification of normal resting state (5 min) and fatigued resting state (5 min), where we treat the above two resting states as separated load states. We argue that after performing the three groups of MATB tasks, the normal and fatigued states might have significant differences that can be easily classified. We adopt a 10-fold cross-validation (CV) to perform the CLR.

Task #2: Four-class classification of normal resting state with Group 1 data, in which we aim to recognize the normal, low, medium, and high loads. We use a 10-fold CV.

Tasks #3 to #5: Three-class classification of low, medium, and high load levels, corresponding with three groups. We aim to recognize the load state during the 5.5-min-long session and observe whether the classification results have a difference. We also use a 10-fold CV. In task #5, the EEG data of subject #13 is removed.

Task #6: Three-class classification to identify low, medium, and high load levels. The training dataset consists of the data of first two groups, while the third group is utilized for testing. We aim to perform cross-session load recognition to verify the time variability. We have removed the data of subject #13 in this task.

The design of the specific load identification task is illustrated in Figure 4. To sum up, tasks #1 to 5 are subject-dependent but have no consideration of time effects, and task #6 considers time variability.

#### 2.4.1 Spectral features

As commonly used, we have adopted power spectrum density (PSD) feature combined with a supporting vector machines (SVM) classifier as a traditional machine learning method. To obtain the time-frequency PSD features of the time-varying EEG data, we have used the short-time Fourier transform (STFT). The concept of employing STFT for processing EEG signals revolves around segregating the signal into distinct frequency bands by adding window functions on the original non-stationary signal and capturing the energy within each band as representative features of the original EEG signals. The five frequency bands are followed with Zhou et al. (2022), by transferring the raw EEG data into delta band [ δ (1–4 Hz)], theta band [ θ (4–8 Hz)], alpha band [ α (8–13 Hz)], beta band [ β (13–30 Hz)], and gamma band [ γ (30–80 Hz)]. Here, we have used the 1-s non-overlap Hanning window. For each sample, the PSD feature dimension across 62 channels is calculated as 62 channels × 2 windows × 5 frequency bands = 620. We have averaged the windowed features to reduce the computing process, and finally use 310 features for each sample.

#### 2.4.2 CNN models

Since CNN models perform better in passive BCI decoding tasks using EEG raw data, we then adopt the widely-used CNN models in the EEG classification domain and transfer them to the load recognition domain. We aim to validate the applicability of CNN models (Schirrmeister et al., 2017; Lawhern et al., 2018; Chen et al., 2024; Ingolfsson et al., 2020) and dig into the load recognition performance. The aim is to offer insights into selecting load-monitoring models. Additionally, we also evaluate and compare the performance of different models, ultimately recommending the one with the highest effectiveness. Detailed structures of these models are presented in Table 2. The model input is shaped as C×T , where C is the number of EEG channels, and T is the number of time points.

**Table 2:** The structures of CNN models used in this paper.

Network	Block	Layer	Filter	Kernel size	Classifier
sCNN	1	Input ( C×T)			Dense (*N*)
		Conv2D-temporal	40	(1, 25)	
	Conv2D-spatial	40	(*C*, 1)		
	AvePooling		(1, 75)		
dCNN	1	Input ( C×T)			Dense (*N*)
		Conv2D-temporal	25	(1, 10)	
	Conv2D-spatial	25	(*C*, 1)		
	MaxPooling		(1, 3)		
	2	Conv2D	50	(1, 10)	
		MaxPooling		(1, 3)	
		Conv2D	100	(1, 10)	
		MaxPooling		(1, 3)	
		Conv2D	200	(1, 10)	
		MaxPooling		(1, 3)	
EEGNet	1	Input ( C×T)			Dense (*N*)
		Conv2D	$F_{1}$	(1, 256)	
	DepthwiseConv2D	$F_{1}*D$	(*C*, 1)		
	AvePooling		(1, 4)		
	2	SeparableConv2D	$F_{2}$	(1, 16)	
		AvePooling		(1, 8)	
EEGNex	1	Input ( C×T)			Dense (*N*)
		Conv2D	$F_{1}$	(1, 32)	
	Conv2D	$F_{1}*4$	(1, 32)		
	DepthwiseConv2D	$F_{1}*4*D$	(*C*, 1)		
	AvePooling		(1, 4)		
	2	Conv2D	$F_{1}*4$	(1, 16)	
		Conv2D	$F_{1}$	(1, 16)	
EEGTCN	1	Input ( C×T)			Dense (*N*)
		Conv2D	$F_{1}$	(1, 64)	
	DepthwiseConv2D	$F_{1}*D$	(*C*, 1)		
	AvePooling		(1, 4)		
	SeparableConv2D	$F_{2}$	(1, 16)		
	AvePooling		(1, 4)		
	2	Conv1D	10	10	
		Conv1D	10	10	

The following are all numbers. C = EEG channels (62), T = time points (500), $F_{1}$ = temporal filters (8), D = depth multiplier in DepthwiseConv2D (2), $F_{2}$ = pointwise filters (16), and *N* = decoded classes, respectively.

The shallow CNN (sCNN) (Schirrmeister et al., 2017) refers to the design of the filter-bank common spatial pattern process and is specially customized for decoding band power characteristics. Specifically, the first two layers of SCNN perform convolution and spatial filtering operations in the time dimension, allowing a wider range of feature extraction and conversion. It has a lighter structure, fewer parameters, and can learn the time structure of power change.

The deep CNN (dCNN) was initially developed to address EEG decoding tasks. It is a model adept at extracting a wide range of features and not confined to specific feature types. Due to its adaptability in the EEG domain, dCNN can effectively handle various EEG classifications, especially when the EEG data exhibits a dispersed distribution.

EEGNet (Lawhern et al., 2018) is a compact CNN that combines deep convolution with separable convolution. It consists of three convolutional layers and one fully connected layer. It aims to capture various EEG feature extraction techniques, such as filter bank construction and optimal spatial filtering. Studies have found that EEGNet exhibits excellent cross-paradigm generalization capabilities and effectively learns a diverse set of interpretable features across various tasks, achieving satisfactory results in EEG classification. The above codes are assessed at https://github.com/vlawhern/arl-eegmodels.

EEGNex (Chen et al., 2024) epitomizes an advanced purely convolution-based neural network architecture derived from EEGNet. It notably enhances the extraction of spatial representations from raw EEG data when compared to the EEGNet. This improvement stems from integrating two 2D convolutions within its overarching structure, utilizing an inverse bottleneck architecture, and expanding the layer’s receptive field. The code can be assessed at https://github.com/chenxiachan/EEGNeX.

EEGTCN (Ingolfsson et al., 2020) effectively leverages temporal information inherent in features and combines EEGNet’s shallow layer as a feature extractor and temporal convolutional network (TCN) to further leverage temporal information. The feature extractor comprises 2D-time convolution, deep convolution, and separable convolution for sequential feature learning and output. Then, TCN extracts temporal information through the integration of time convolution blocks and causal convolutions and a 1D fully convolutional network, ultimately feeding into the fully connected layer. The code can be assessed at https://github.com/iis-eth-zurich/eeg-tcnet.

We have adopted the widely used BCI methods for MATB task-based load recognition. For tasks 1 to 5, we use a 10-fold CV to evaluate the recognition performance. Task 6, can be seen as the fixed train-test design to evaluate the cross-time or cross-session generalization capability. Overall, all the tasks can be considered as subject-dependent strategies to validate findings and recognition performance.

The performance metrics are accuracy and *F*_1_ score. The traditional method that uses an SVM classifier combining PSD features is implemented in Matlab 2019a. The CNN-based models are implemented with the Tensorflow 2.5.0 library in PyCharm 2020.1.2 and Python 3.7.0. The CNN-based models were trained for 50 epochs using a batch size of 32. The Adam optimizer was employed, and the learning rate was set to 0.0001.

## 3 Experimental results

This section includes the subjective analysis of the NASA TLX scale, the objective operate-related behavior data analysis, and the EEG load recognition results under different classifiers and six CLR tasks.

### 3.1 Subjective analysis

The NASA-TLX scale comprises six subscales: mental demand, temporal demand, physical demand, effort, performance, and frustration (Hart, 1988). Participants were instructed to rate each subscale on a scale from 0 to 100. The final results were derived as the weighted average of these subscales.

**Figure 5:**
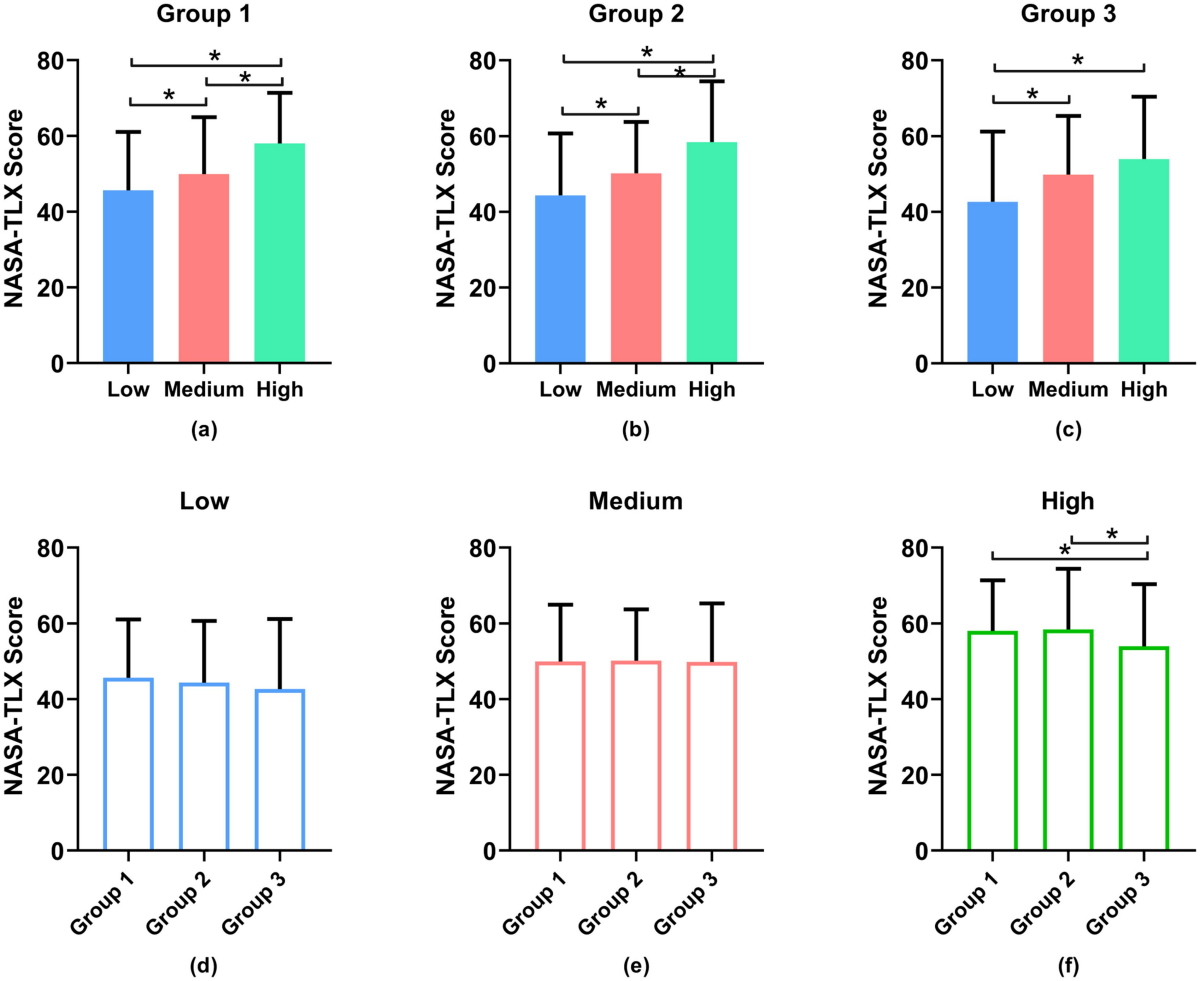
Subjective results, with **(a–c)** across different groups and **(d–f)** across time. *Denotes statistically significant (*p* > 0.05).

Figure 5 depicts the average NASA-TLX scores of all participants at different task difficulty levels during different periods, using paired t-tests for statistical analysis. The analysis of variance results reveals a significant increase in NASA-TLX scores among participants as task difficulty rises (*p* < 0.05). More specifically, in Figures 5a,b, a notable distinction (*p* < 0.05) can be observed in the subjective ratings across the three difficulty levels during the first and second sessions (Groups 1 and 2). However, in the third session (Group 3), the difference between the moderate and high difficulty levels was not significant (*p* > 0.05).

We also compared three load levels across different groups, as illustrated in Figures 5d–f. Though the presentation order of Groups 2 and 3 is different from Group 1, the subjective scale comparison results indicate that the difference between the low and medium load levels among the three groups was not significant (*p* > 0.05). Similarly, there is no significant difference between the first and second groups under high load, but a significant difference was observed between the first two groups and the third group (*p* < 0.05). This suggests that the randomization of load levels within each group is justified.

### 3.2 Objective results

In the formal experiment, after each set of MATB tasks is completed by the participants, the MATB software automatically records the objective data for subsequent analysis. We then computed three objective metrics: response time for the system monitoring task, tracking error in the tracking task, and standard deviation of fuel level in the AB tanks in the resource management task.

**Figure 6:**
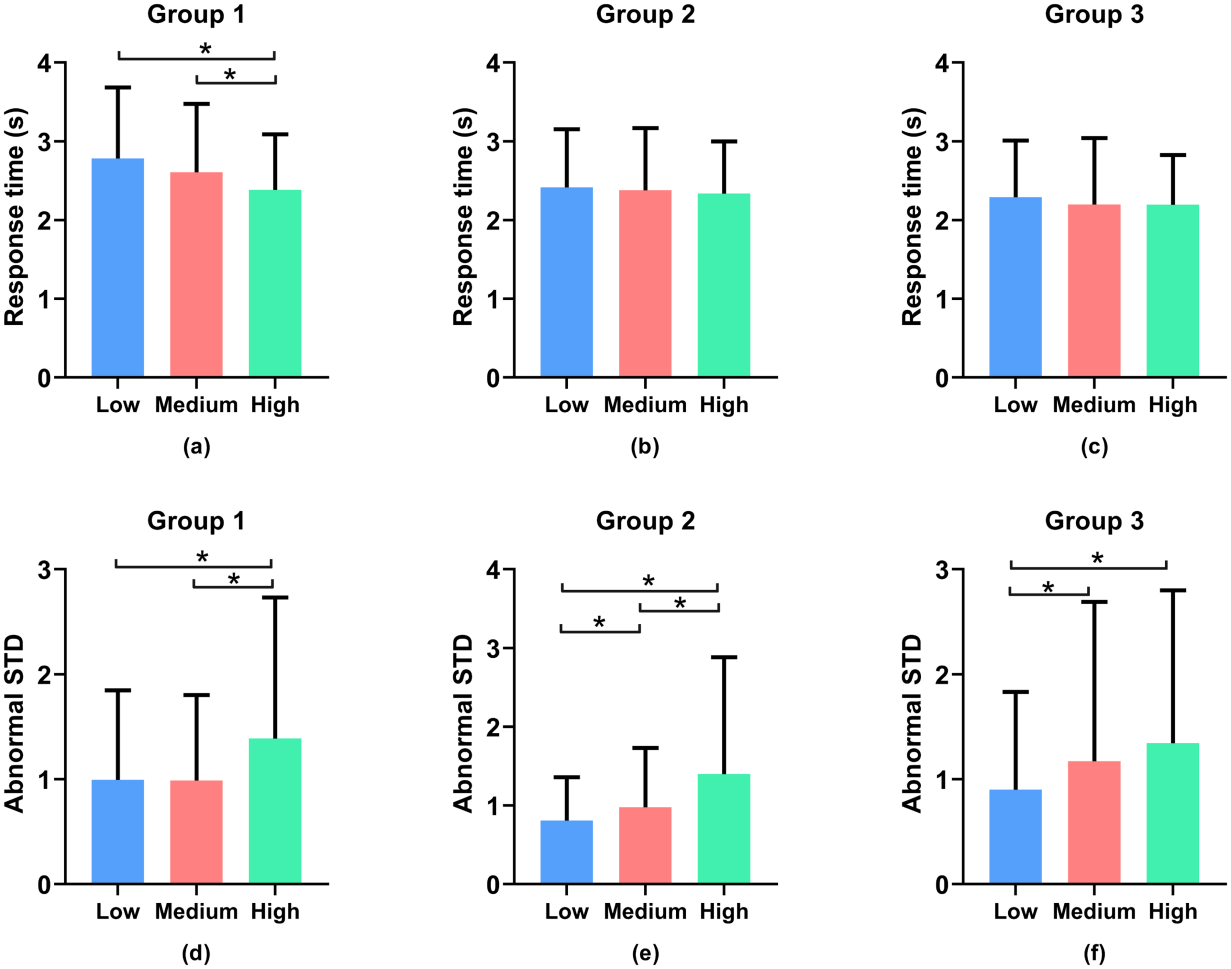
Objective task performance, including response time for the system monitoring task **(a–c)** and tracking error in the tracking task **(d–f)**. *Denotes statistically significant (*p* > 0.05).

Figure 6 presents the average objective task performance, including response time for the system monitoring task (the first row) and tracking error in the tracking task (the second row), for all participants across the three sessions. The operator’s response time in the system monitoring task is relatively insignificant. As seen in Figure 6a, the response time of low load state versus high, and medium versus high, are significantly different (*p* < 0.05). In Groups 2 and 3 (Figures 6b,c), the operator’s response time in the system monitoring task remains insignificant (*p* > 0.05). The graph clearly illustrates that as task difficulty increases, there is a significant increase in tracking error (computed as abnormal STD) in the tracking task. For example, as shown in Figures 6d–f, the low load state has significantly lower error than the high load states, the low load state is significantly different from medium states in Groups 2 and 3, the medium load state is significantly different with high states in Groups 1 and 2 (*p* < 0.05). These results indicate a decreasing trend in task performance as the difficulty level increases.

**Figure 7:**
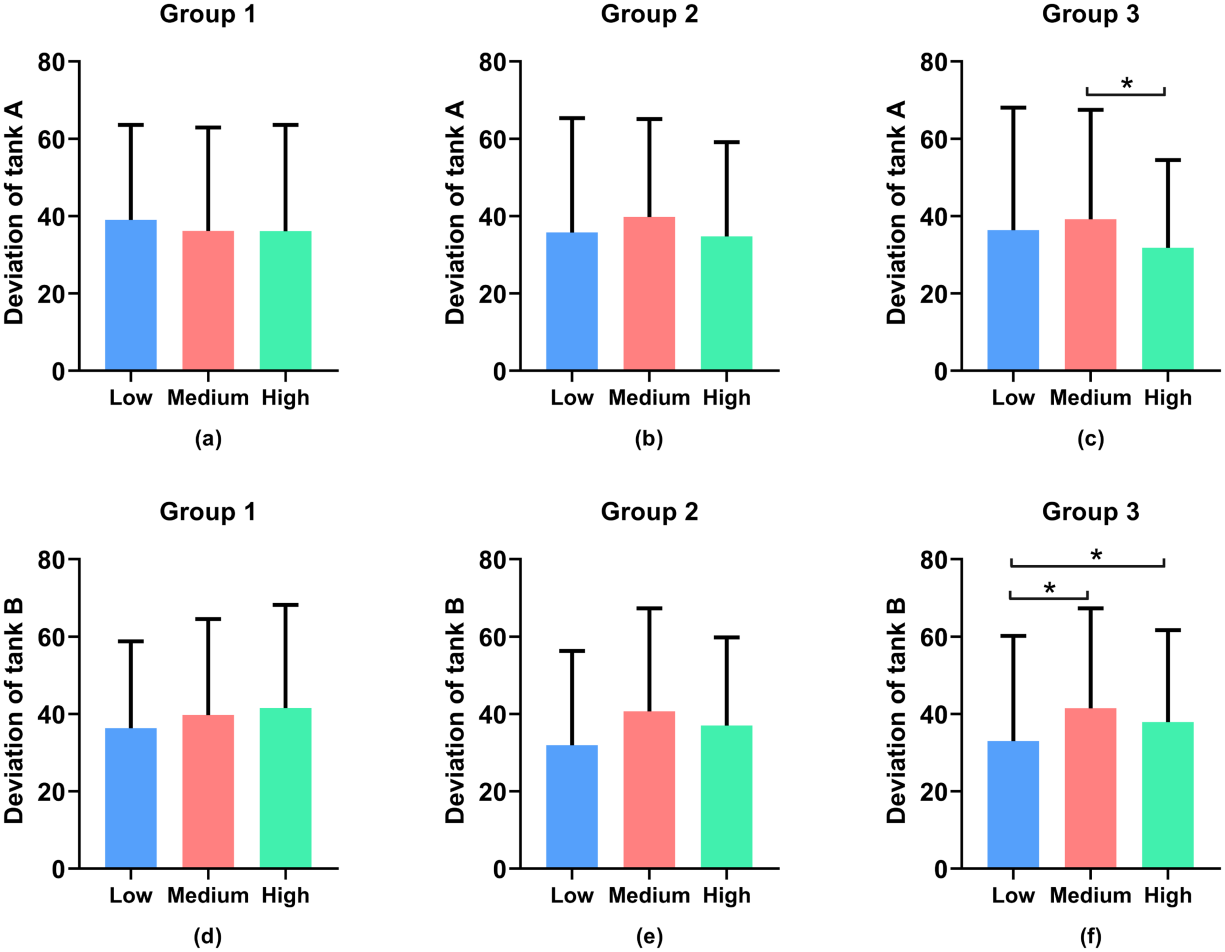
Objective results of the standard deviation of fuel level in the resource management task, where **(a–c)** are for tank A and **(d–f)** are for tank B. *Denotes statistically significant (*p* > 0.05).

In Figures 7a,b,d,e, in Groups 1 and 2, the standard deviation of fuel level in the resource management task is not statistically significant (*p* > 0.05), while in Figures 7c,f, it is significant. Overall, these findings demonstrate that the designed tasks can effectively elicit varying levels of cognitive load among participants, as evidenced by both the subjective rating scale and the objective task performance data.

### 3.3 Classification results

**Figure 8:**
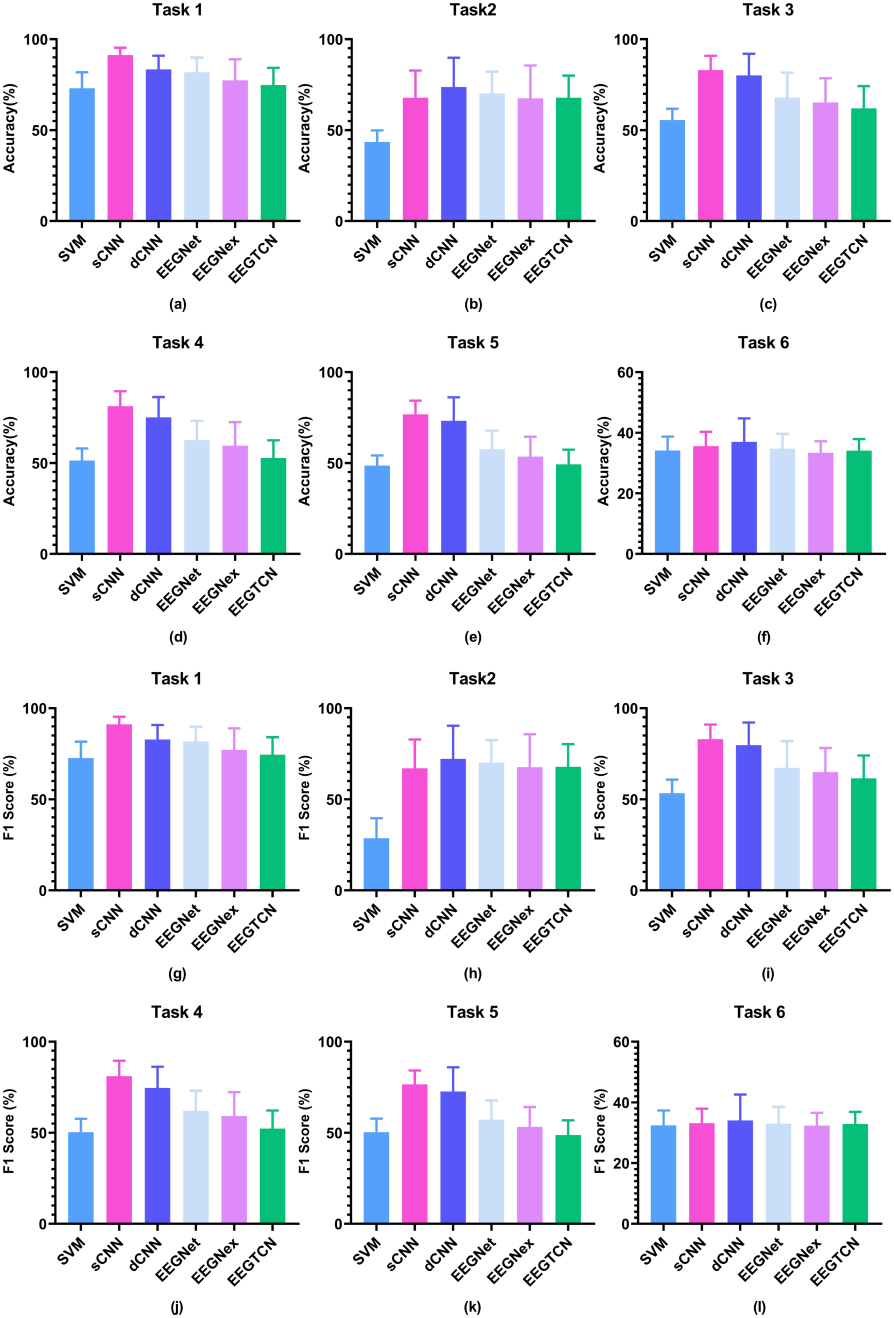
CLR results under six tasks, where **(a–f)** are accuracy results and **(g–l)** are *F*_1_ score results.

The CLR results of six tasks are shown in Figure 8. In task 1, the CNN model outperforms the traditional PSD + SVM method (with an accuracy of 73%). Among them, the sCNN model exhibits the best performance (with an accuracy of 91.3%). Other models include dCNN (accuracy of 83.39%), EEGNet (81.81%), EEGNex (77.38%), and EEGTCN (74.78%).

In task 2, the CNN model outperformed the traditional PSD + SVM method (with an accuracy of 43.53%). Specifically, the dCNN model achieved the best accuracy of 73.64%, followed by the EEGNet (70.27%), sCNN (67.78%), EEGTCN (67.77%), and EEGNex (67.49%).

In task 3, the CNN model outperforms the PSD + SVM (with an accuracy of 55). Specifically, the shallow CNN model achieved the best accuracy of 83%. Additionally, the other models were the dCNN (with an accuracy of 80%), EEGNet (67.95%), EEGNex (65.26%), and EEGTCN (61.99%).

In task 4, the CNN model consistently outperforms the PSD + SVM approach (accuracy of 51%). Among them, the shallow CNN model demonstrates the highest accuracy (81%). Other models include the dCNN (accuracy of 75%), EEGNet (62.58%), EEGNex (59.52%), and EEGTCN (52.77%).

In task 5, the CNN model outperforms the PSD + SVM approach (with an accuracy of 48.51%). Specifically, the shallow CNN model demonstrates the best performance (with an accuracy of 76.74%), followed by dCNN (73.19%), EEGNet (57.63%), EEGNex (53.52%), and EEGTCN (49.27%).

When applied to task 6, namely within-subjects cross-time load classification, the traditional method, and CNN-based models exhibited poorer recognition performance than tasks 3 to 5, and these three methods have similar performance. Specifically, in task 6, the traditional PSD + SVM method achieved an accuracy of 34.13%, with the dCNN model exhibiting the best performance with an accuracy of 36.97%. Other models included sCNN (accuracy of 35.61%), EEGNet (34.7%), EEGTCN (34.11%), and EEGNex (33.40%).

To sum up, comparing the traditional PSD + SVM classifier with the CNN-based models, we can observe that (1) the CNN models have better decoding performance, and (2) the simpler the CNN model, the more adept it becomes at decoding when the input is an original signal.

## 4 Discussion

The discussion section has several parts. The sample size and accuracy of each individual are shown first. Second, model convergence is shown. Third, ablation research is carried on. Fourth, visualization of learned feature representation is displayed. Finally, the limitations are discussed.

### 4.1 Brain activation pattern

**Figure 9:**
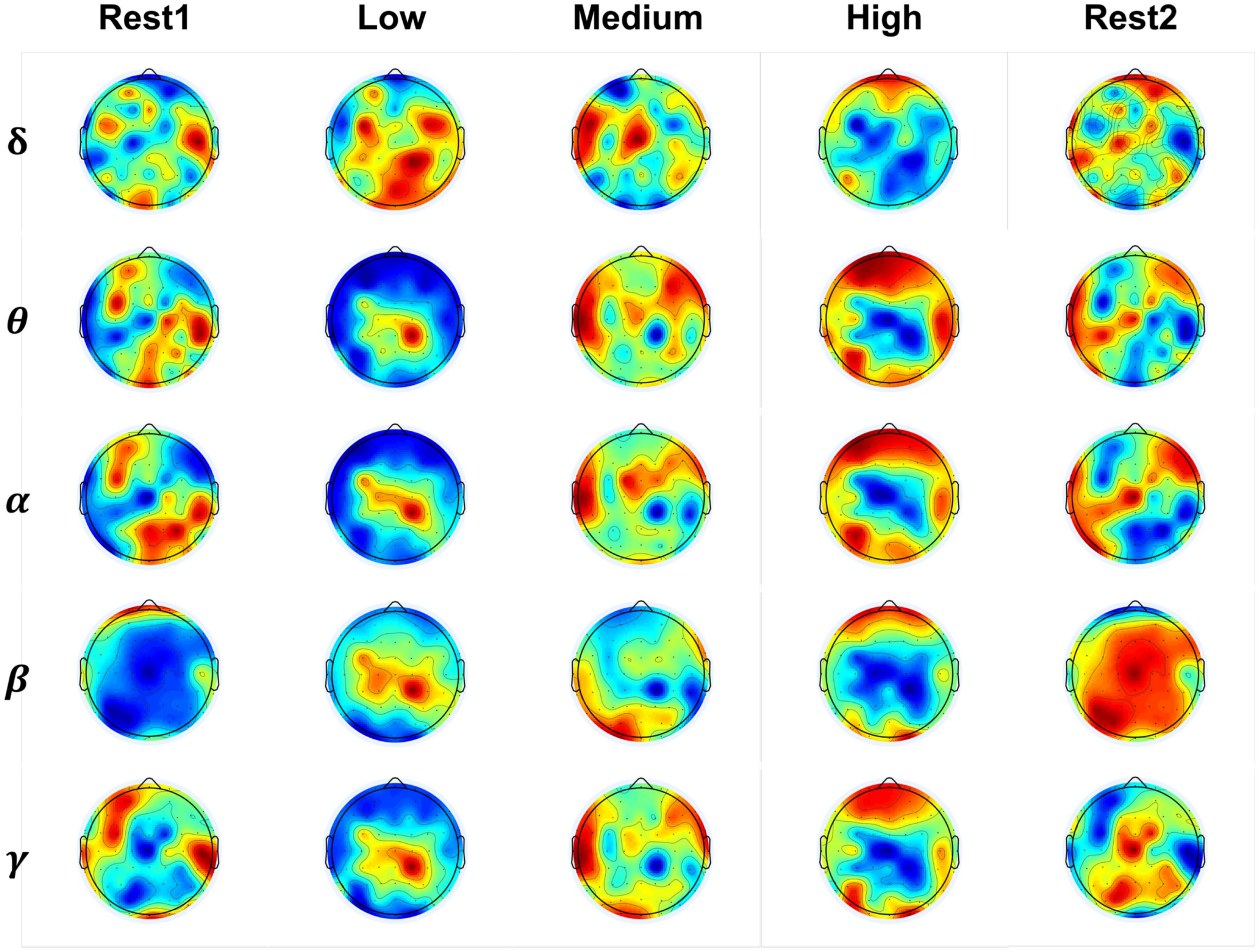
The brain activation patterns across various frequency bands and cognitive states. Red indicates positive activation, blue represents negative activation, and the intensity of the color corresponds to the magnitude of the activation value.

We present the PSD distribution of EEG across various frequency bands and cognitive states to compare brain activation patterns, with five frequency bands. By calculating the average PSD across 36 subjects for each task state and normalizing it to [0, 1], we obtain the PSD matrix for each state. This matrix is then converted into a channel-by-frequency band format for visualization. The PSD distribution results are displayed in Figure 9.

The β band and α band have been reported they be associated with the load changes (Dimitrakopoulos et al., 2017; Zhou et al., 2022), indicating the brain is in engaged and high conscious states, respectively. Focusing on the two frequency bands, we can see there is similar brain pattern activation in both resting states; the difference lies in whether the activation is positive or negative. By comparing the colors, it can be observed that the positive activation value in the fatigued resting state is larger (more intense red) in the left temporal and central brain regions than in the non-work resting state. Between the high load state to the low load states, the activation pattern of PSD features is more elicited in the frontal, occipital and parietal brain regions.

Besides, we have used the paired *t*-test to make the statistical analysis between the different load states. The statistical analysis is given in Table 3 with *p* < 0.05. Between the resting state 1 and fatigued resting state 2, there are significant differences in three frequency bands except the low-frequency δ band and high-frequency γ band. Between the low-load state and medium-load states, there are significant differences in all four frequency bands except the low-frequency δ band. Between the low load state and high load states, there are significant differences in all four frequency bands except the high-frequency β band. Between the medium load state and high load states, there are significant differences in δ band and β band.

**Table 3:** Statistical analysis of brain activity patterns across various load conditions.

Bands	Rest1 vs. Rest2	Low vs. medium	Low vs. high	Medium vs. high
δ	\\	\\	**	*
θ	**	**	**	\\
α	**	**	**	\\
β	**	**	\\	*
γ	\\	**	**	\\

\\ means there is no significant difference, and * denotes the significant difference with *p* < 0.05, and ** denotes the significant difference with *p* < 0.01.

### 4.2 Time effects

Additionally, we examined how the decoding model influenced the subjects’ operational states over time by comparing the decoding performance across the three model groups. In this analysis, data from subject #13 were omitted from the experimental evaluation, focusing on the results from 35 subjects in the sCNN model.

**Figure 10:**
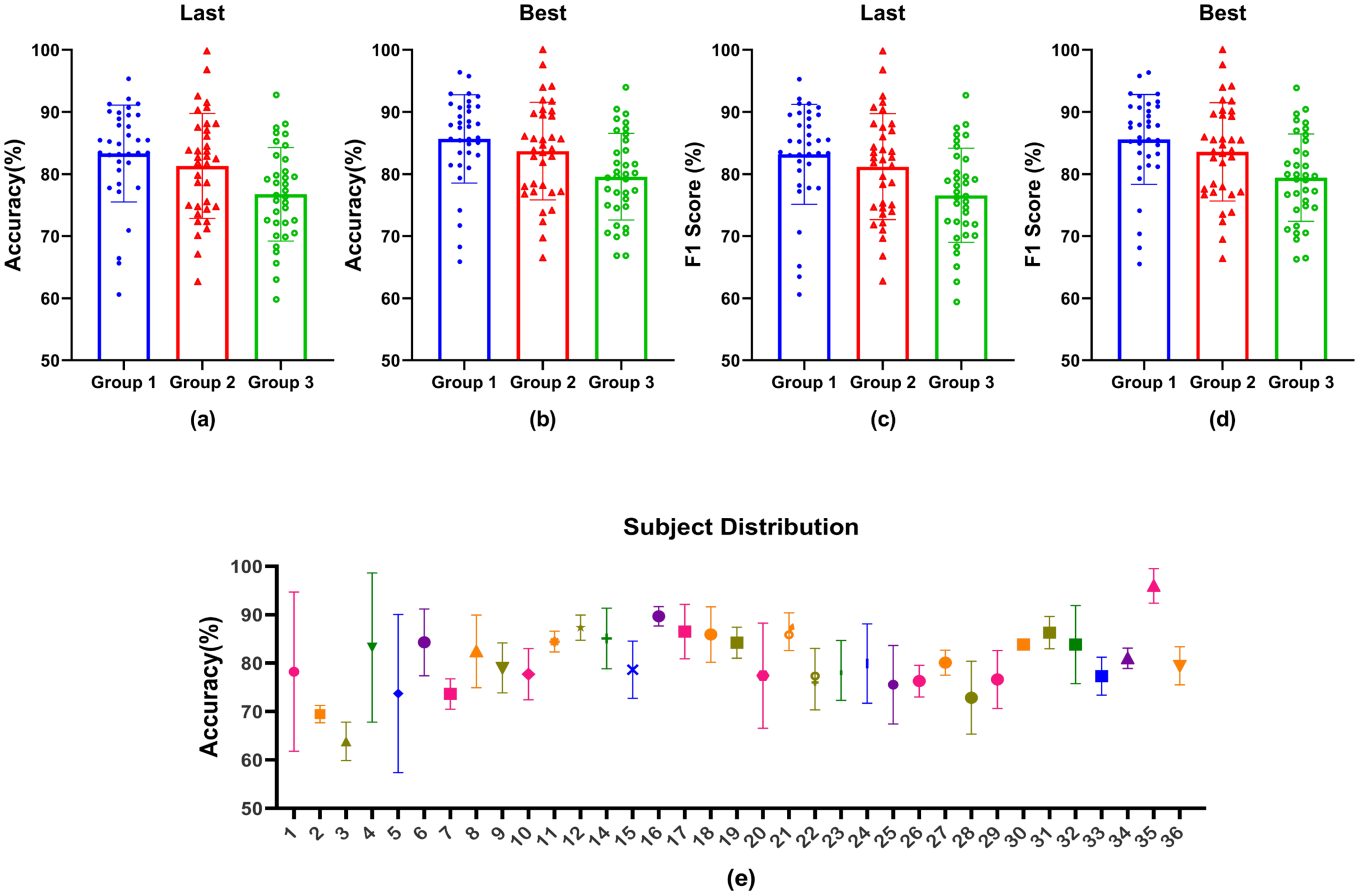
The impact of the decoding model on the working state of subjects over time, **(a,c)** denote last results, **(b,d)** denote the best results, and **(e)** is the accuracy distribution across subjects.

Figures 10a,c represent the average classification accuracy and F1 score of the sCNN model across tasks 3 to 5 (i.e., Groups 1 to 3). Here, “Last” denotes that we take the result from the 50th epoch as the final result for each subject. Additionally, we conducted experiments to document the best classification metric observed, subsequently averaging these values to obtain Figures 10b,d with “Best.” The results in Figures 10a–d indicate that recognition performance has significantly decreased tendencies over time. Comparing Figure 10a vs. Figure 10b and Figure 10c vs. Figure 10d, the best condition has better results than the last one.

Figure 10e illustrates the accuracy distribution across subjects. Subjects # 1, 4, 5, and 20 exhibited notable variations in their accuracy outcomes across the three groups, potentially due to fatigue incurred by these individuals throughout the high-cognitive-load experiment. Of all the subjects, subjects #16 and 35 demonstrated consistent and superior performance on cognitive tasks, whereas subjects #2 and 3 exhibited inferior performance.

### 4.3 Feature visualization

**Figure 11:**
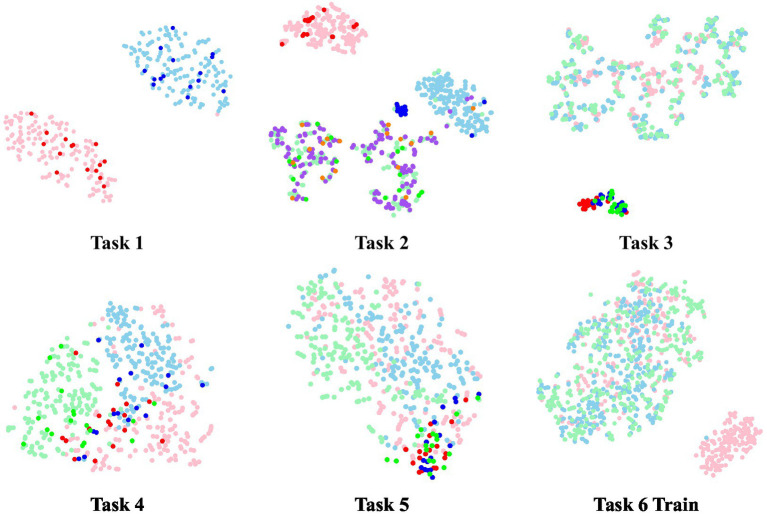
The t-SNE visualization of Sub #A.

**Figure 12:**
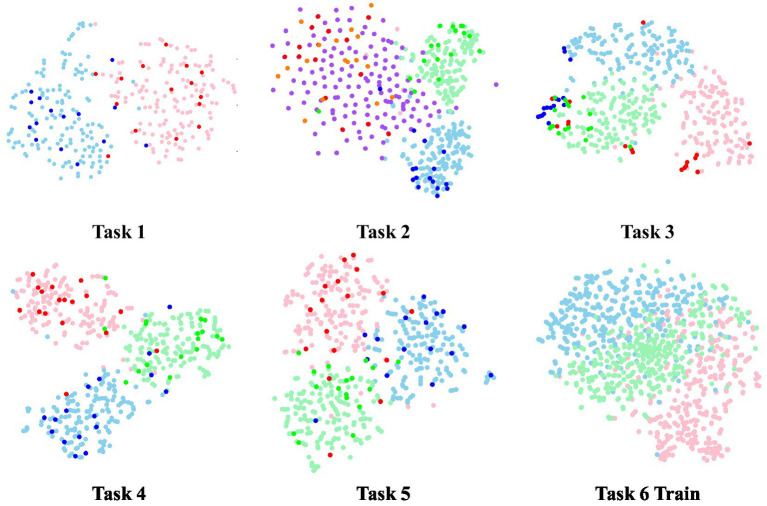
The t-SNE visualization of Sub #B.

The study utilizes t-distributed Stochastic Neighbor Embedding (t-SNE) (van der Maaten and Hinton, 2008) to map the feature representation onto a 2D plane for visualization, as depicted in Figures 11, 12. t-SNE is a nonlinear dimensionality reduction technique designed to maintain the relationships within the data in a lower-dimensional representation. Two subjects were randomly chosen, and the feature distribution of both training and test data was visualized after the first-fold of model training, with the respective task number displayed at the bottom. In the case of task 6, only the training features were visualized. Light colors indicate features from the training data, while dark colors represent features from the test data. For instance, in task 1, red signifies normal resting state data and blue signifies fatigue resting state data. Similarly, in tasks 3, 4, 5, and 6, pink represents low load data, blue represents medium load data, and green represents high load data.

It is evident that distinguishing task 1 in subject A is relatively straightforward, as features within the same cluster are well-integrated and features of the same class align well between training and testing data. While the resting state and low load of task 2 can be differentiated, medium load and high load samples lack a clear classification boundary, resulting in significant overlap. The cognitive load classification of different data groups reveals challenges in distinguishing tasks 3 and 5 (Groups 1 and 3), with some distinguishability in task 4 with Group 2 data. The distribution results of task 6 indicate difficulty across time in distinguishing training data.

Similar to subject A, in Figure 12, task 1 in subject B is easily distinguishable, with consistent characteristics within the same class across training and testing data. Task 2 can be separated into low and medium loads. Tasks 3, 4, and 5 are distinguishable from subject A, with data from different load states forming distinct clusters. However, distinguishing the distribution data for task 6 is challenging.

Comparing the above two subjects, subject B has a better BCI adaptation and discriminatory than subject A, which also indicates the variation between subjects.

### 4.4 Limitations and future outlook

This study has several limitations. Firstly, the narrow time interval of this study does not encompass a broad period, potentially impeding a comprehensive understanding of cognitive load changes. Secondly, the recognition performance in cross-time decoding was suboptimal than within-subject design, failing to effectively capture the dynamic EEG changes over time.

Future research can focus on, first, developing an efficient deep model tailored for cognitive load decoding to enhance performance across periods, such as the model that integrates CNN and self-attention (Song et al., 2023). Furthermore, we can use interpretability tools to explain the spatiotemporal patterns learned by CNNs (e.g., specific frequency bands or brain regions). Second, exploring advanced transfer learning algorithms (Shao et al., 2024; Zhou et al., 2022; Zhou et al., 2023) that mitigate time and subject variability, thereby bolstering the robustness and adaptability of decoding models to cognitive load changes. For example, in our previous work, to ease the issue of high subject variance in CLR, we have proposed a domain adaptation model for joint shallow and deep feature alignment (Zhou et al., 2023). The model incorporates a distributional difference measure to adjust shallow feature distributional offsets, and one domain discriminator to reduce inter-domain distributional differences. Further, we have introduced the bi-classifier joint domain adaptation (BCJDA) model (Shao et al., 2024) tailored for cross-session and cross-subject CLR, incorporating domain-level and class-level alignment. These models reduce the distributional differences between different subjects or periods, thereby facilitating the effective utilization of multi-subject shared features for the mining of subject/time stability patterns and the construction of models for subject generalization. The advanced deep decoding model for efficient feature learning and transfer learning methods that can reduce the EEG variance will constitute the focus of the next step of our research.

## 5 Conclusion

This study aimed to investigate the evaluation criteria for cognitive load state in a complex operational task environment, with a specific focus on methods utilizing EEG signals and behavior data. Our results suggest that, when exposed to prolonged cognitive load, participants exhibit heightened sensitivity towards high-load states, displaying behavioral reactions and brain activation patterns that differ significantly from those observed in low-load states. Specifically, a stronger activation of high-frequency EEG was observed in states of high load. Additionally, the results of our classification study indicate shallow CNN models yield superior recognition results in within-subject decoding tasks, which is noteworthy. Our study provides further confirmation that cognitive load states are substantially influenced by temporal factors in continuous cognitive tasks, as evidenced by the downward trend in recognition performance observed between the groups. Besides, all models demonstrate inadequate performance in cross-time generalization, thereby necessitating future research to focus on time variation and subject discrepancy. In conclusion, this paper establishes a potential foundation for assessing cognitive states in intricate simulated MATB tasks across different individuals. Future research may delve deeper into the application of deep CNN models in cross-session and cross-individual decoding, aiming to enhance the precision and dependability of cognitive load state evaluation.

## Data Availability

The original contributions presented in the study are included in the article/supplementary material, further inquiries can be directed to the corresponding author.
